# Role of Basal ABA in Plant Growth and Development

**DOI:** 10.3390/genes12121936

**Published:** 2021-11-30

**Authors:** Benjamin P. Brookbank, Jasmin Patel, Sonia Gazzarrini, Eiji Nambara

**Affiliations:** 1 Department of Cells and Systems Biology, University of Toronto, Toronto, ON M3S 3G5, Canada; ben.brookbank@mail.utoronto.ca (B.P.B.); jas.patel@mail.utoronto.ca (J.P.); 2 Department of Biological Sciences, University of Toronto Scarborough, Toronto, ON M1C 1A4, Canada

**Keywords:** ABA, chloroplast biogenesis, hyponastic growth, skotomorphogenesis, stomata, cutin, wax, suberin, xylem, hormone crosstalk, ethylene

## Abstract

Abscisic acid (ABA) regulates various aspects of plant physiology, including promoting seed dormancy and adaptive responses to abiotic and biotic stresses. In addition, ABA plays an im-portant role in growth and development under non-stressed conditions. This review summarizes phenotypes of ABA biosynthesis and signaling mutants to clarify the roles of basal ABA in growth and development. The promotive and inhibitive actions of ABA in growth are characterized by stunted and enhanced growth of ABA-deficient and insensitive mutants, respectively. Growth regulation by ABA is both promotive and inhibitive, depending on the context, such as concentrations, tissues, and environmental conditions. Basal ABA regulates local growth including hyponastic growth, skotomorphogenesis and lateral root growth. At the cellular level, basal ABA is essential for proper chloroplast biogenesis, central metabolism, and expression of cell-cycle genes. Basal ABA also regulates epidermis development in the shoot, by inhibiting stomatal development, and deposition of hydrophobic polymers like a cuticular wax layer covering the leaf surface. In the root, basal ABA is involved in xylem differentiation and suberization of the endodermis. Hormone crosstalk plays key roles in growth and developmental processes regulated by ABA. Phenotypes of ABA-deficient and insensitive mutants indicate prominent functions of basal ABA in plant growth and development.

## 1. Introduction

The sesquiterpenoid hormone abscisic acid (ABA) regulates numerous plant processes, including growth and development [1]. ABA plays a well-characterized role in promoting seed and bud dormancy, as well as regulating stress responses in various plant species [2,3,4]. During dormancy establishment and following stress, ABA levels sharply increase leading to inhibition of germination and growth arrest to mount a stress response. However, in well-watered plants, basal amounts of ABA are required to promote growth and development of various organs and tissues [5,6]. Here, we summarize the phenotypes of ABA biosynthesis and signaling mutants and highlight the role of basal ABA in plant growth and development as well as its interaction with other hormones, with a focus on genetic evidence.

### 1.1. Everything in Moderation: ABA Deficiency and over-Accumulation

ABA-deficient mutants are useful for elucidating its roles in plant growth and development. Phenotypes of ABA-deficient mutants depend on the severity of ABA deficiency and the types of enzymes mutated. ABA is synthesized via oxidative cleavage of 9-cis-epoxycarotenoids derived from β-carotene [7]. Thus, mutants defective in β-carotenoid biosynthesis and its downstream pathway show ABA deficiency. Due to pleiotropic phenotypes of carotenoid deficiency, mutants defective in zeaxanthin epoxidase (ZEP) or more downstream enzymes are used for ABA-deficient mutants in research. ZEP catalyzes a two-step epoxidation of zeaxanthin to violaxanthin. The *zep* mutants have been isolated from various plants, including Arabidopsis *aba1*, tomato *high-pigment 3* (*hp3*), and rice *osaba1* [8,9,10]. The *zep* mutants over-accumulate zeaxanthin and show decreased levels of epoxycarotenoids and downstream metabolites, including ABA [8,10]. Zeaxanthin and epoxycarotenoids play an important role in quenching reactive oxygen species (ROS), byproducts of photosynthesis, to protect photosynthesis proteins from photo-oxidative damage [11]. Thus, phenotypes of *zep* mutants are caused by both ABA deficiency and altered xanthophyll contents. On the other hand, mutants of 9-cis-epoxycarotenoid dioxygenase (NCED), xanthoxin dehydrogenase (ABA2) and abscisic aldehyde oxidase (AAO3) are thought to be the specific ABA-deficient mutants. Severity of ABA deficiency is often associated with the number of gene family members. The Arabidopsis *AtABA2*, rice *OsABA2* and pea *PsABA2/WILTY* are single-gene coded in the genome, therefore mutations in these genes show severe ABA deficiency [12,13]. NCEDs are encoded by multigene families, so the severity of ABA deficiency largely depends on expression divergence among family members. The well characterized *nced* mutants include maize *vp14*, Arabidopsis *nced* mutants, tomato *notabilis* (*not*) [7]. Also, inhibitors to block the NCED enzyme activities, such as nordihydroguaiaretic acid (NDGA), abamine SG and hydroxamic acid inhibitor D4, have been used to chemically induce ABA deficiency [14,15,16]. AAO3 catalyzes the last step of ABA biosynthesis. Arabidopsis *aao3* and tomato *sitiens* (*sit*) have been well characterized for various physiological responses [7]. In Arabidopsis, *AAO3* seems to be the only aldehyde oxidase (AO) encoding the physiological abscisic aldehyde oxidase, but other three AOs convert abscisic aldehyde to ABA at a basal level in the *aao3* mutant due to the promiscuity of substrate recognition [17]. Thus, the severity of ABA deficiency of the *aao3* mutant is less pronounced compared to the *aba2* mutant. AAO3 is an AO that requires a molybdenum cofactor (MoCo), thus mutants having a defect in MoCo biosynthesis show ABA deficiency. In plants, nitrate reductases (NR), xanthine dehydrogenases (XDH), peroxisomal sulfite oxidases (SO) and AOs are well characterized as MoCo containing enzymes in plants [18]. MoCo sulfurase encoded by ABA3 in Arabidopsis catalyzes the maturation step of MoCo biosynthesis required for AO and XDH function. Thus, Arabidopsis *aba3* and tomato *flacca* (*fla*) mutants have defects in AO and XDH activities [19,20]. Therefore, the *aba3* mutant shows both ABA-dependent and independent phenotypes [21]. On the other hand, barley Az34 mutant show defects in NR, XDH, as well as AO indicating that the genetic defect of this mutant is upstream of ABA3, causing more pleiotropic phenotypes than the *aba3* mutant [22].

ABA is inactivated by oxidation and sugar conjugation [7]. CYP707As, a subfamily of P450 monooxygenases, catalyzes ABA 8′-hydroxylation responsible for oxidative catabolism of ABA. ABA glycosylation inactivates ABA, but ABA glucose ester is stored and can be released by ABA β-glucosidase (BG). The *cyp707a* mutants contain higher ABA levels, which decrease slower when dehydrated plants are watered or dry seeds are imbibed [23,24,25]. Selective inhibitors for CYP707As have been developed by utilizing P450 inhibitors or ABA analogs as lead compounds [26,27]. These inhibitors are useful to maintain endogenous ABA at the high levels, although the right choice of concentrations is required to minimize off-target effects [28].

### 1.2. Perception Is Key: ABA Insensitivity and Hypersensitivity

The ABA signaling pathway requires three types of proteins as core components: PYR/PYL/RCAR receptors, group A protein phosphatase 2C (PP2C) and Sucrose Non-Fermenting 1-Related Protein Kinase 2 (SnRK2), [1]. PYR/PYL/RCAR receptors inhibit the phosphatase activity of PP2Cs in the presence of ABA [29,30]. Inhibition of PP2C, negative regulators of ABA signaling, derepresses SnRK2 to induce the downstream events [31]. Mutants of these core signaling genes are thought to be specific ABA signaling mutants [32,33]. In addition, transcription factors (TFs) identified from forward genetic analysis, such as ABA-INSENSITIVE3 (ABI3), ABI4 and ABI5 are important downstream regulators of the core signaling pathway [1,34]. Genes encoding the core signaling proteins belong to multigene families. For example, the Arabidopsis genome encodes 14 ABA receptor genes, while the rice genome contains 13 ABA receptor genes. Therefore, phenotypes of ABA receptor mutants are often visible only when higher order mutants are constructed [30,35,36,37]. Group A *PP2C* and *SnRK2* form smaller gene families, therefore single mutants often show ABA-specific phenotypes. Six out of nine Group A PP2Cs are characterized as negative regulators of ABA signaling in Arabidopsis [38]. Loss-of-function mutants for *ABA-INSENSITIVE1* (*ABI1*) and *ABI2* are hypersensitive to ABA [39]. Mutants defective in *ABA HYPERSENSITIVE GERMINATION 1* (*AHG1*) and *AHG3* show a pronounced ABA hypersensitive phenotype during seed germination [40]. Mutations in the catalytic domain of PP2C, such as *abi1-1*, *abi2-1* and *hab1^G246D^* confer dominant ABA-insensitive phenotypes [41]. Subclass III SnRK2 kinases are positive regulators of ABA signaling. Five out of 10 Arabidopsis SnRK2s are activated by ABA in a short period of time [42]. In particular, SnRK2.2/SRK2D, SnRK2.3/SRK2I and SnRK2.6/SRK2E/OST1 are primarily responsible for ABA signaling [38]. Antagonists and agonists against ABA receptors have been reported, which are also useful to induce ABA related phenotypes in plant species that are difficult to conduct genetic analysis [27,43,44].

## 2. Role of ABA in Plant Growth

ABA-mediated growth regulation involves crosstalk with other hormones and nutritional signaling, to regulate various aspects of cellular growth, including cell division, enlargement, differentiation, and central metabolism. Our understanding of how ABA regulates cellular growth is still fragmental. In rice, low concentrations of ABA are shown to upregulate a wide array of cell cycle related genes, including Cyclin-dependent kinases (CDKs) and cyclins (CYCs), in addition to auxin-related biosynthesis, transport, and signaling genes [45]. ABA regulation of cell cycle is in part explained by crosstalk with auxin and cytokinins (CK), both of which are involved in cell cycle regulation [46,47]. ABA has been shown to promote CK signaling via ABI4 [48]. ABI4 binds to the promoter of type-A ARRs and supresses their transcription. Type-A ARRs serve as negative feedback regulators of CK signaling. Furthermore, type-A ARRs are upregulated in ABA-deficient mutants. CKs critically regulate cell division by promoting progression through key cell cycle checkpoints. CK application induces the expression of CDKs and CYCD proteins involved in both G1/S and G2/M phase transitions [49,50,51,52]. Cell elongation involves several mechanisms and is correlated with endoreduplication [53]. Endoreduplication occurs when cells enter an endocyclic phase. The endocycle is an alternative type of cell cycle where mitosis is skipped, resulting in repeated DNA replications within one nucleus (reviewed in [54]).

**Figure 1:**
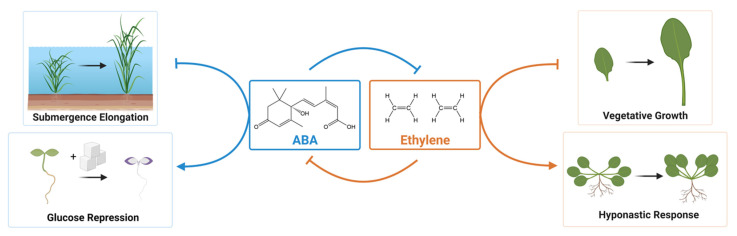
Regulation of plant growth by ABA and Ethylene antagonism. ABA and ethylene function in a mutually antagonistic way to regulate various aspects of plant growth. A prerequisite for shoot elongation in rice is ABA catabolism, which requires ethylene action. High concentrations of glucose are known to result in the growth arrest of Arabidopsis seedlings. This arrest depends on ABA synthesis and signaling and is antagonized by ethylene. Ethylene accumulation is thought to inhibit vegetative growth. One key role of basal ABA is to inhibit ethylene biosynthesis in growing plants. Ethylene signaling promotes hyponastic responses in Arabidopsis, which are antagonized by ABA. Figure created with BioRender (https://biorender.com/, accessed on 27 November 2021).

ABA is recognized as a growth inhibitor. This view is justified based on two lines of evidence: (i) high concentrations of exogenously applied ABA result in growth arrest [55] and (ii) endogenous ABA accumulates under a variety of stress conditions, concomitant with a reduction of growth in the stressed plant. This evidence ties in neatly with the identity of ABA as a stress hormone; since plants must balance growth programs against stress responses, it follows that ABA signaling should tip the scale in favour of allocating resources to cope with adverse environments at the expense of growth. However, the mode of ABA-mediated growth regulation varies depending on concentrations, timing, and tissues, showing both positive and negative effects on growth [5]. Context-dependent positive and negative growth regulation is known for other hormones, such as ethylene, CK and auxin [56,57,58]. This coincides with the prominent role of hormone crosstalk in ABA-mediated growth. We will summarize ABA-mediated growth regulation with an emphasis on the phenotypes of ABA-deficient and insensitive mutants (see also Figure 1 and Table 1).

### 2.1. Halt! Who Grows There? Growth Inhibition by Basal ABA

The outcome of basal ABA in growth inhibition should be seen as growth promotion in ABA-deficient and insensitive mutants. Growth promotion of these mutants is observed in local tissues or under particular conditions. Hyponastic growth is the upward bending of leaves that involves growth promotion of cells at the abaxial side. This growth is an adaptive strategy of plants to changing environments, such as submergence and shade avoidance [100]. In Arabidopsis, ABA-deficient (*aba1*, *aba2*, *aba3*) and ABA-insensitive (*abi1*, *abi3*) mutants show enhanced hyponastic growth of petioles, which are visible as increases in petiole angles [59]. The petiole angle of the wild type decreases when exogenous ABA is applied. Whereas the application of fluridone, an inhibitor of phytoene desaturases to block metabolite accumulation upstream of ABA biosynthesis, increases the petiole angle. The local growth inhibition by ABA at the petiole is antagonistic to ethylene, which promotes hyponastic growth [59] (Figure 1). It is noteworthy that different Arabidopsis accessions display differential growth responses to these hormones [59]. In Rumex, hyponastic growth is a critical avoidance response to complete submergence [101]. Submergence-induced ABA decreases are a critical step to elongate the shoot in flooding tolerance [102]. The decrease of ABA under submergence is a prerequisite for ethylene and gibberellin action [101]. The shoot growth of *osaba1* is enhanced compared with wild type when rice seedlings are submerged [99]. Submergence-induced rapid decrease of ABA is associated with the induction of *OsABA8ox1* encoding ABA 8′-hydroxylase [99]. Importantly, submergence-induced ABA decrease is ethylene dependent (Figure 1). Recently, Toriyama et al. reported that a moss RAF-like kinase orthologous to Arabidopsis *CONSTITUTIVE TRIPLE RESPONSE1* (*CTR1*) is required for both ethylene-induced submergence response and ABA-dependent SnRK2 activation [103]. These collectively indicate ABA-ethylene interaction in submerged plants are at both metabolism and signaling levels.

Some ABA-deficient and insensitive mutants of rice show enhanced growth. Rice *osaba2* mutants defective in the xanthoxin dehydrogenase are viable in standard conditions. Interestingly, the plant heights of these mutants are taller than wild type, which is a typical and expected phenotype of mutants defective in growth inhibitors [13]. Although showing excessive growth, the *osaba2* mutants do not appear to be healthy. These mutants were identified as a lesion mimic mutant showing spontaneous cell death with an overaccumulation of reactive oxygen species (ROS). The phenotype of *osaba2* mutants indicates basal ABA plays a role in suppressing ROS production and growth under normal conditions. Some multigenic mutants of rice ABA receptors show increased growth compared to wild type [36]. The rice genome encodes 13 ABA receptors structurally divided into two groups: group I and II [36]. Miao et al. (2018) found that a particular combination of multigenic mutants from Group I receptors grows better and produces greater yield than wild type. Thus, the role of ABA in growth and stress tolerance is genetically separable at the signaling level in rice. In contrast, ABA-hypersensitive Arabidopsis lines such as the PP2C triple mutants *hab1 abi1 abi2* and *hab1 abi1 pp2ca* show reduced size compared to wild type [80]. Stunted growth of ABA hypersensitive mutants agrees with the role of ABA in growth inhibition. This phenotype is readily explained by a constitutive activation of stress-related ABA genes, facilitating a stress response even in non-stressful environments [80].

One explanation for the stunted growth of ABA-related mutants is an increased sensitivity to environmental factors. Mutants are exposed to stresses more sensitively than wild type due to a lack of stress resistance mechanisms. ABA-deficient mutants of tomato grow faster and taller with an increased number of leaves when grown under mist [84]. Similarly, Arabidopsis ABA-deficient *aba2* mutant and *snrk2* triple mutants show enhanced growth on agar plates, which are relatively humid compared to standard soil conditions [67]. Excess growth of *aba2* and *snrk2* triple mutants is associated with increased respiration through the tricarboxylic acid cycle [67]. In ABA-deficient mutants of both tomato and Arabidopsis, a characteristic aspect of growth enhancement is the increase in the leaf numbers [67,84]. Initiation of leaf formation is a well-controlled process and used for determining biological time for flowering. It is particularly interesting to understand the mechanism of how basal ABA regulates leaf initiation. Interestingly, the increased leaf number was not observed in an ABA-insensitive quadruple *areb* mutant, suggesting that the ABA signaling to negatively regulate leaf initiation is SnRK2-dependent, but independent of AREB-mediated transcription [67].

High concentrations of exogenous ABA inhibit lateral root (LR) growth in Arabidopsis [104]. Ethylene and auxin act downstream of ABA in the regulation of Arabidopsis root elongation [104]. Arabidopsis ABI4 AP2 TFs play an important role in inhibiting LR growth [82]. The *abi4* mutant enhanced LR formation and growth, which is characterized by increased LR density and elongated LRs. This mechanism involves counteracting ABA and CK action to regulate expression of *ABI4*, which in turn disturbs auxin transport [82]. As mentioned above, tomato *sit* and pea *wilty* mutants increase LR, indicating low concentrations of ABA can inhibit the formation of LR [94]. The mechanism for ABA-mediated inhibition of LR growth is dynamic and alters depending on time. The inhibition of wild-type LR growth is alleviated by a prolonged ABA treatment, while this recovery is delayed in the *pyl8* mutant [105]. This indicates that PYL8 is required for the recovery of LR growth from ABA inhibition in a prolonged period. This involves the PYL8-MYB77 interaction to induce auxin-responsive genes independently of SnRK2 [105]. Consistent with this mechanism, application of auxin rescued the delayed recovery phenotype of the *pyl8* mutant [105].

### 2.2. Debunking a Myth: Growth Promotion by ABA

Unsurprisingly, there are ample reports indicating a role for ABA in growth promotion [5]. In this section, we summarize the negative effect of ABA deficiency and insensitivity on growth. The promotive effect of ABA on growth should be observed as smaller plants when ABA biosynthesis or signaling is genetically blocked. Importantly, applying low concentrations of exogenous ABA rescues the stunted growth of ABA-deficient mutants [68,85]. ABA-deficient Arabidopsis and tomato mutants show a stunted shoot growth and reduced fresh weight compared to wild type [60,65,77,95]. Furthermore, ABA-insensitive Arabidopsis lines such as higher order *pyl112458* sextuple, *pyl* duodecuple mutants and *snrk2.2 snrk2.3 snrk2.6* mutants also display the dwarfed plant phenotype [32,35,37]. In agreement with this, Arabidopsis plants overexpressing SnRK2.6 were shown to have an increase in biomass compared to wildtype [106]. One cause of the stunted growth in ABA-related mutants is due to an overproduction of ethylene (Figure 1). Ethylene overproduction is observed in ABA-deficient mutants of tomato and Arabidopsis [69,85,86]. Blocking ethylene action by genetically introducing ethylene insensitivity partially reverts the stunted shoot growth of these ABA-deficient mutants [68,69,85]. This indicates that a role of basal ABA in the promotion of growth is to negatively regulate ethylene biosynthesis. The same mechanism is also applicable to maize root growth under low water potential [98]. Importantly, the authors conclude that ABA-mediated ethylene suppression is only part of its effect on vegetative growth; there also exists an ethylene-independent role for ABA.

A positive role of ABA in the primary root growth is to properly maintain the root meristem. This involves two modes of action; inhibiting cell division of quiescent centre (QC) and suppressing stem cell differentiation, both of which are required for proper meristem functions [61]. Both ABA-deficient (*aba1*, *aba2*, *aba3*) and insensitive (*abi1-1*, *abi2-1*, *abi3* and *abi5*, but not *abi4*) mutants show enhanced cell division in QC. Importantly, the treatment of an ABA biosynthesis inhibitor fluridone also enhances cell division in QC, which is reversed by co-application of a low concentration of ABA [61]. A pharmacological experiment suggests that ABA-mediated inhibition of QC cell division is independent of ethylene, although ethylene promotes cell division in QC [61].

Grafting experiments indicate that shoot-derived ABA regulates root growth that involves inhibition of LR development and increased root to shoot ratio [94]. The roots of ABA-deficient mutants have increased indole-3-acetic acid (IAA) levels, suggesting the function of ABA to promote root growth involves the decrease the root auxin levels [94]. In Arabidopsis, the regulation of root elongation by exogenous ABA is biphasic. Low concentrations of exogenous ABA promote root growth, while high concentrations inhibit root growth [107].

Hypocotyl growth is achieved solely by cell elongation [108]. The hypocotyl elongates in the dark, while its growth is inhibited in the light. This dark-induced mode of growth is termed skotomorphogenesis. Humplik et al. (2015) reported that etiolated seedlings of *sit* and *not* mutants show short hypocotyls [87]. These phenotypes are rescued by applying low doses of exogenous ABA, suggesting that these processes are associated with the growth promotion by ABA in darkness. The short hypocotyl phenotype of Arabidopsis ABA-deficient mutants is also rescued by the application of low concentrations of ABA [62]. In these tomato and Arabidopsis mutants, authors discuss a defect in cell differentiation or faulty induction of cell expansion. Tomato ABA-deficient mutants show decreased expression of *SlKRP1* and *SlKRP3*, blocked endoreduplication, and elevated CKs. In Arabidopsis, ABA induces the expression of *ICK1/KRP1*, which is thought to antagonize CDKA [109]. In plants impaired in ABA biosynthesis or signal transduction, endoreduplication is affected, resulting in reduced cell expansion. This may be a cause of the reduced rosette size in ABA-deficient and insensitive lines.

ABA promotes fruit growth and maturation. Fruit ripening is categorized into two types; climacteric and non-climacteric, based on the nature of respiration and ethylene dependence. ABA promotes both climacteric and non-climacteric fruit ripening [110,111]. In tomato, a climacteric fruit, ripening is accelerated by knock-down of a negative regulator of ABA signaling, *SlPP2C1*, or by overexpressing an ABA receptor, *SlPYL9* [112,113]. Pharmacological experiments demonstrate that ABA promotes tomato fruit ripening via induction of ethylene synthesis [114]. Consistently, *SlPP2C1* RNAi lines show earlier ethylene production [112]. On the other hand, *flc* and *flc not* mutants produced smaller fruits [86]. This indicates that ABA promotes fruit growth. Interestingly, fruits of the severe ABA-deficient *flc not* double mutant, but not the *flc* single mutant, over-produce ethylene. This indicates that basal ABA blocks ethylene over-production, which is opposite to the induction of ethylene synthesis by ABA signaling reported in Zhang et al. (2018) [112]. Thus, crosstalk between ABA and ethylene can be positive or negative, depending on the type of tissue or stage of development studied.

Aside from stomatal-dependent gas exchange influencing photosynthesis, ABA regulates central metabolism and nutritional signaling, such as carbon metabolism and sugar signaling. Genetic screening for glucose-mediated inhibition of Arabidopsis seedling growth identified *aba2* and *abi4* mutants as glucose insensitive [70]. This indicates that glucose mediated growth inhibition requires ABA biosynthesis and ABI4 function. This ABA-mediated growth inhibition is antagonized by ethylene signaling [89] (Figure 1).

ABA regulates carbon metabolism and transport that impacts growth. High concentrations of ABA mimic stress conditions, which negatively regulate photosynthesis and carbon assimilation. This is supported by down-regulation of nuclear and chloroplast encoded photosynthesis genes by exogenous ABA [1,115,116]. Also, ABA application promotes accumulation of soluble sugars [117]. ABA Moreover, overexpression of apple *MdAREB2* induces expression of *MdSUT2*, a sucrose transporter gene, via direct binding to its promoter [118]. On the other hand, the role of basal ABA on photosynthesis is controversial, possibly depending on the context. Tomato *sit* mutants show an increased net assimilation rate when compared with wild type under non-stressed conditions [92]. Tomato *fla* and barley Az34 both have a defect in MoCo biosynthesis, which decreased net assimilation rates [93]. Moreover, it is differentially affected by relative humidity in which net assimilation of Az34, but not *fla*, is negatively affected by low humidity. The Arabidopsis *aba1* mutants show a decreased photosynthesis (PSII) activity [8].

One characteristic phenotype of ABA-deficient mutants is a defect in chloroplast biogenesis. Arabidopsis *aba1* mutants show an increased number of chloroplasts with aberrant ultrastructure [8,10]. The *aba1* mutants over-accumulate zeaxanthin and disturb the xanthophyll content, which may affect chloroplast biogenesis and function. The increase of chloroplast numbers and its aberrant ultrastructure can also be seen in the tomato *high-pigment3* (*hp3*) mutant defective in *ZEP*. Interestingly, similar defects in chloroplast biogenesis can also be seen in *fla* and *sit* mutants of tomato [10]. These indicate that basal ABA, rather than zeaxanthin over-accumulation, is required for maintaining proper chloroplast biogenesis and functions. It will be interesting to investigate how disturbed chloroplast biogenesis impacts plant growth as well as sugar and energy metabolism in ABA-related mutants under both non-stressed and stressed conditions.

## 3. Role of ABA in Plant Development

**Figure 2:**
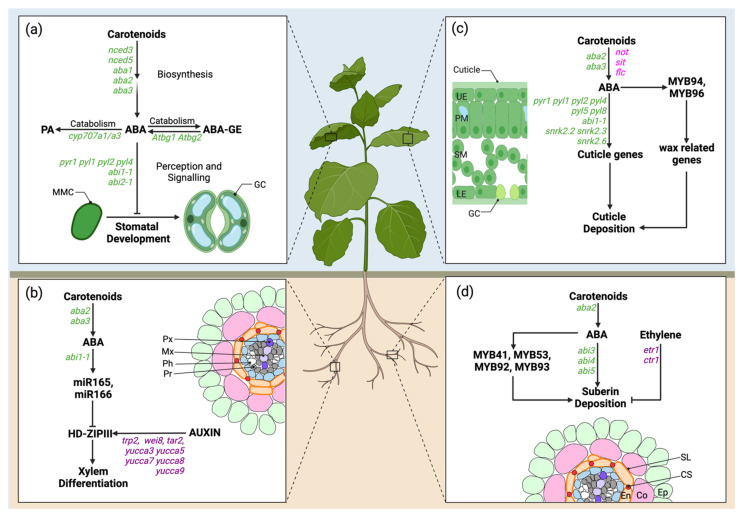
Overview of the role of basal ABA in stomata development, cuticle and suberin deposition, and xylem differentiation. Genetic analysis of ABA synthesis and signalling demonstrates a role for basal ABA in stomatal development, protoxylem differentiation, and deposition of cuticle and suberin. (**a**) ABA biosynthetic and signalling mutants show increased stomatal index or density, while ABA catabolism mutant display decreased stomatal index or density, indicating a negative role for ABA in stomatal development. (**b**) ABA promotes protoxylem differentiation through upregulation of *miR165/166* in the endodermis, which downregulates *HD-ZIP III* in the stele. Xylem differentiation is disrupted in ABA biosynthesis and signaling mutants, which develop discontinuous metaxylem strands. (**c**) ABA promotes cutin deposition, by indirectly regulating wax and cutin-related genes. In Arabidopsis and tomato ABA biosynthetic mutants, hydrophobic cutin and wax layers are reduced. In Arabidopsis, ABA signaling mutants display increased cuticle permeability. (**d**) ABA positively regulates suberin deposition. ABA biosynthetic and signaling mutants show decreased suberin accumulation. ABA regulates several MYB TFs, which in turn promote suberin or cuticle deposition. Other hormones also regulate these processes: ABA regulates suberin accumulation antagonistically to ethylene, while acting in parallel with auxin in xylem differentiation. ABA related mutants are shown in green for Arabidopsis and in magenta for tomato; ethylene and auxin mutants are shown in lilac/purple. Panels (**a**) and (**c**) abbreviations: MMC, meristemoid mother cell; GC, guard cell; UE, upper epidermis; PM, palisade mesophyll; SM, spongy mesophyll; LE, lower epidermis. Panels (**b**) and (**d**) abbreviations and legend: epidermis (Ep), light green; cortex (Co), light pink; endodermis (En), light orange; suberin lamellae (Sl), orange; Casparian strip (Cs), red; pericycle (Pe), light blue; procambium (Pr), grey; phloem (Ph), white; protoxylem (Px), purple; metaxylem (Mx), light purple/lilac. Figure created with BioRender (https://biorender.com/, accessed on 27 November 2021).

To adapt their growth under a changing environment, land plants have developed specialized cells and tissues that are optimized for gas exchange and water transport, while minimizing water loss and pathogen attacks. In aerial organs, a layer of hydrophobic compounds, mainly cutin and waxes, cover the epidermis to prevent water loss and protect from pathogens, while stomata, which are epidermal pores, control water and gas exchange [119]. Below ground, hydrophobic compounds such as suberin and waxes surround the mature endodermis of the roots, to control water and nutrient movements from the soil into the xylem vessels of the stele [120]. Here, we review the role that basal ABA plays in the development of these tissues under non-stressed conditions (see also Figure 2 and Table 1). The role of ABA in seed and plant dormancy, somatic embryogenesis, senescence and fruit ripening has been recently reviewed and will not be covered here [2,3,121,122,123].

### 3.1. Breath-Taking: ABA Inhibition of Stomatal Development

Aside from its well-known function in regulating stomata physiology and promoting stomata closure [124], ABA also plays an important role in stomata development. ABA levels inversely correlate with stomata density (number of stomata per unit of area) and index (percentage of stomata cells to the total number of epidermal cells). Genetic analysis using ABA metabolism mutants has revealed that ABA inhibits entry of epidermal cells into the stomata development pathway. Several Arabidopsis ABA-deficient mutants (*aba1*, *aba2*, *aba3* and *nced3 nced5*), including mutants with reduced levels of active ABA (*Atbg1 Atbg2*), display increased stomatal density or index. In contrast, ABA catabolism mutants (*cyp707a1 cyp707a3*) show decreased stomata density or index [63,64,66,72,83]. Similarly, tomato ABA-deficient *flc*, *sit* and *not* mutants have increased stomata density and index [88]. The increased stomata density of ABA biosynthetic mutants can be rescued by application of ABA [72,125] or by expressing ABA biosynthetic genes only in stomata lineage cells, suggesting a cell autonomous ABA-dependent regulation of stomata development [66]. ABA also decreases guard cell size and is required for pavement cell expansion, suggesting ABA regulates the final size of epidermal cells [72]. Perturbation of the ABA signaling pathway also affects stomata development; the dominant, ABA insensitive *abi1-1* and *abi2-1* mutants display increased stomata index [64,72]. Thus, ABA and a functional ABA signaling pathway are required to limit entry of epidermal cell into the stomatal lineage pathway (Figure 2).

In Arabidopsis, stomata development is promoted by the sequential action of three basic helix–loop–helix TFs, SPEECHLESS (SPCH), MUTE and FAMA. The stomatal development pathway is repressed in non-stomatal lineage cells by a family of leucine-rich repeat receptor kinases and coreceptors (TMM, ER, ER-like, SERK). Activation of the receptors by the peptide ligands, EPIDERMAL PATTERNING FACTOR1 (EPF1) and EPF2, repress SPCH through phosphorylation by a MAP kinase signaling cascade [75,126]. In Arabidopsis, mutations that decrease the levels of active ABA (*Atbg1 Atbg2*) correlate with upregulation of *SPCH*, *MUTE* and *FAMA* [83]. In *aba2*, which was shown to prolong the development of meristemoids and guard mother cell in cotyledons, the expression of *SPCH* and *MUTE* is also extended, suggesting a temporal effect of ABA in restricting entry into the stomatal development pathway [72]. However, in *nced3 nced5*, expression level of *SPCH* and *MUTE* was not affected, while *EPF2* transcript level was increased, possibly due to the increased number of stomata precursor cells or feedback regulation [66]. Genetic interaction studies between *aba2* and *spch* or *mute* indicate that ABA inhibits stomatal development at the level of *SPCH* [72]. These findings demonstrate that in the absence of stress, ABA-mediated inhibition of stomatal development acts through the well-characterized stomatal development pathway (Figure 2).

Stomatal development is regulated by environmental signals, including light, water, temperature, and CO_2_ (Reviewed in [75,126,127]). ABA biosynthetic (*nced3 nced5* and *aba3*) as well as perception (*pyr1 pyl1 pyl2 pyl4*) mutants fail to decrease stomatal density in response to elevated CO_2_. This phenotype can be complemented by targeted expression of *ABA3* or *NCED3* in *aba3* or *nced3 nced5* stomata lineage cells, respectively, suggesting a cell autonomous mode of action [66]. Thus, inhibition of stomatal development by elevated CO_2_ requires ABA as well as a functional ABA signaling pathway in guard cells or its precursors.

Manipulation of CK, auxin, ethylene and BR levels or sensitivity also affects stomata density or patterning mainly acting at the level of SPCH, whose function is well conserved across different plant species [128]. These studies have shown that ABA, jasmonates, and auxin inhibit while CKs and ethylene promote stomata development. In contrast, brassinosteroids (BR) promote stomata development in hypocotyls, while inhibiting stomata formation in cotyledons [63,90,91,129,130,131,132,133,134,135]. MAPK integrates ABA and BR signaling in the control of stomatal development. While ABA activates MKKK20-MKK5-MPK6 cascade by sequestering ABI1 phosphatase, which promotes phosphorylation and degradation of SPCH, negative regulation of BIN2 by BR activates YDA-MKK4-MKK5 cascade, which results in phosphorylation and degradation of SPCH [131,136]. Crosstalk between several hormone signaling pathways ensures that multiple signals are integrated to regulate stomata development. However, the mechanism by which hormone signaling pathways interact to regulate stomatal development through SPCH is currently unclear.

### 3.2. You Shall Not Pass! The Role of ABA in Cutin and Waxes Deposition

The cuticle is a hydrophobic layer covering the epidermis of land plant aerial organs. The cuticle is composed of cutin, an aliphatic polyester, and waxes, which are secreted and deposited on the outer side of the plant cell wall. The cuticle acts as a layer of protection against environmental stress, primarily preventing non-stomatal water loss and pathogen attacks, as well as diffusion of materials across the cell wall [119,137].

Physiological, molecular, and genetic studies have shown that basal ABA promotes the synthesis and deposition of cutin and waxes [71,96,97,138,139,140]. ABA increases wax content in the cuticle by inducing expression of *MYB94* and *MYB96*, which can directly bind to and positively regulate wax biosynthetic genes [139,140]. Furthermore, the cuticles of ABA-deficient mutants (*aba2* and *aba3*) display increased permeability and ROS production in Arabidopsis [71]. Similarly, the leaves of tomato ABA-deficient mutants (*not*, *sit*, *flc*) show reduced expression of genes involved in cutin and wax formation, resulting in decreased levels and altered composition of cutin and cuticular waxes [96,97]. These phenotypes were rescued by application of ABA and resemble those displayed by cuticle biosynthesis mutants (*bdl* and *lacs2*), indicating that ABA biosynthesis is required to promote cuticle development even in the absence of stress [71,96,97] (Figure 2).

Mutations affecting core components of the ABA signaling pathways (*pyr1 pyl1 pyl2 pyl4 pyl5 pyl8*, *abi1-1*, and *snrk2.2 snrk2.3 snrk2.6*) also result in increased cuticle permeability [78]. Several cuticle-related genes encoding for biosynthetic enzymes and transporters, as well as positive (MYB94, MYB96) and negative (DEWAX) regulators of wax synthesis and cutin deposition are differentially regulated in ABA metabolic (*aba3*, *cyp707a1 cyp707a3*) or signaling (*snrk2.2 snrk2.3 snrk2.6*, *abf2 abf3 abf4*, and *abi1-1*) mutants [78,141]. However, mutations in ABA signaling components downstream of SnRK2 (*abf2 abf3 abf4*) did not show defects in cuticle permeability, suggesting ABA signaling may indirectly regulate cutin deposition through activation of MYBs and negative regulation of DEWAX [78]. Wax-related genes, including CER6, have ABA-responsive elements in their promoters and are induced by ABA, thus it is possible that ABA directly regulates wax synthesis (Reviewed in [142]) (Figure 2). In the moss *Physcomitrella patens*, ABA negatively regulates the expression of cuticle-related genes, suggesting different evolution of ABA function during cuticle deposition in flowering and non-flowering plants [78].

Abiotic and biotic stresses alter the thickness and composition of the cuticle in several species. While increased cuticle thickness promotes tolerance to abiotic stresses such as drought, altered composition and amount of cuticle have different effects during plant interaction with pathogens; in some cases it promotes plant tolerance to biotic stresses, while in others it has an inhibitory effect (reviewed in [143]). Cutin also mediates plant response to osmotic stress by regulating ABA biosynthesis. Following osmotic stress, mutants impaired in cutin biosynthesis show reduced induction of ABA biosynthesis genes leading to decreased ABA accumulation and reduced tolerance to osmotic stress, although the mechanism is unclear [144]. Thus, not only does cutin play a protective role against environmental stress, but it is also required for osmotic stress induction of ABA biosynthesis. By regulating cuticle deposition, ABA plays different roles in plant response to biotic and abiotic stresses.

### 3.3. Xylem Differentiation: ABA and Auxin Act in Concert

Water transport from the root to the shoot is mediated by the xylem. Made of cellulose, hemicellulose and lignin, the secondary cell wall (SCW) is deposited in the xylem and provides support and strength to withstand the negative pressures of water transport. In Arabidopsis roots, the protoxylem is composed of spiral or annual SCW and develops at the periphery of the stele, while the metaxylem is made of reticulated or pitted walls and forms in the center of the stele [145]. Xylem identity is specified by the interplay between class III homeodomain leucine zipper (HD-ZIP III) TFs and microRNA165 (miR165)/miR166. miR165/miR166 are expressed in the endodermis and move to the xylem to restrict HD-ZIP III expression pattern in a dose-dependent manner, with protoxylem specified by low HD-ZIPIII level in the periphery and metaxylem by high level of HD-ZIPIII in the middle of the stele [146,147].

Recent studies have shown that ABA is required for SCW deposition and specification of xylem identity. In Arabidopsis and tomato roots, exogenous ABA induces early protoxylem differentiation at the root tip as well as protoxylem formation in place of metaxylem further away from the root tip. In contrast, ABA deficiency, induced either genetically (*aba2*, *aba3*) or chemically (fluridone), disrupts xylem development and results in the formation of discontinued metaxylem strands [73,74]. Furthermore, impaired ABA perception and signaling mutants (*abi1-1*, *pyr1 pyl1 pyl2 pyl4*) fail to induce ectopic protoxylem in the presence of ABA [73,74]. Mechanistically, ABA induces *miR165A*/*166B* through the core ABA signaling pathway. Targeted inhibition of ABA signaling in the endodermis, but not in the stele, is sufficient to inhibit xylem differentiation via *miR165* induction [73,74] (Figure 2). These studies show that ABA acts in the endodermis and upstream of miR165/166-HD-ZIP III module to promote protoxylem differentiation in the stele in a non-cell autonomous manner. Interestingly, reduced ABA signaling in the epidermis or columella can also affect xylem differentiation, implying the existence of another non-cell autonomous ABA-dependent mechanism [73,74]. A similar switch in xylem cell identity is also observed in roots exposed to osmotic and water stress, which elevate endogenous ABA levels [73,74]. Under water stress, an increase in miR165 restricts *PHABULOSA (PHB)* expression, leading to an increase in protoxylem development and a switch from a pitted to a reticulate metaxylem [73]. In maize, embolism induced by water stress was more frequent in metaxylem compared to protoxylem, and an increase in the number and thickness of vessels was observed also in other plant species subjected to water stress, suggesting that this developmental switch may confer an advantage under low water conditions [148,149,150,151].

Auxin, CKs, JA and BR also regulate vascular patterning and xylem differentiation in the stele (Reviewed in [145]). In the stele, auxin biosynthesis mutants (*trp2*, *wei8 tar2*, quintuple *yucca*) show defects in metaxylem development [152], while inhibition of auxin signaling completely inhibits xylem development; the latter can be partially restored by exogenous ABA, suggesting ABA and auxin promote xylem differentiation through independent pathways [74]. In the stele, auxin biosynthesis is required for the induction of *HD-ZIP III* genes [152], which bind to the promoter of *MP*, a master regulator of vascular development, and its negative regulator *IAA20*, to regulate metaxylem formation [153]. These studies show that ABA and auxin regulate xylem differentiation independently: ABA induces *miR165A* in the endodermis, whereas auxin activates *HD-ZIP III* in the stele (Figure 2).

In the inflorescence stem, ABA was shown to promote SCW deposition, which is controlled by a set of NAC TFs, including NAC SECONDARY WALL THICKENING PROMOTING FACTOR 1 (NST1)/ANAC043. Genes related to SCW synthesis are downregulated in inflorescence stem of ABA-deficient (*aba2*) and signaling (*snrk2.2 snrk2.3 snrk2.6*) mutants. Accordingly, these mutants have thinner cell walls and decreased lignin and crystalline cellulose content [81]. ABA regulation of SCW formation requires phosphorylation of NST1 by SnRK2s. Interestingly, the SnRK2-mediated phosphorylation site is conserved in NST1 orthologs in dicots, but not in monocots, suggesting different regulatory mechanisms controlling SCW deposition in monocots and dicots [81]. Overexpression of *NCED6* and *NCED9*, which induce the expression of SCW-related genes, did not increase lignification and SCW deposition, suggesting ABA alone is not sufficient to promote SCW deposition.

### 3.4. A Corky Story: Antagonistic Role of ABA and Ethylene in Suberin Deposition

Suberin is a phenolic and aliphatic heteropolymer that can be found in various tissues including cork, seed coats, tree bark, root periderm and root endodermis in higher plants. In the endodermis, suberin lamellae are deposited to create SCW that acts to shift the endodermis from an actively absorbing tissue to a protected endodermis. This action depends on the developmental stage as well as hormone signals that are based off environmental stimuli [76].

Several studies have shown that exogenous application of ABA induces the expression of suberin-related genes and increases suberin deposition in the endodermis, while triggering ectopic suberin deposition in root tips and in potato tubers [76,154,155,156,157,158,159]. An essential role of basal ABA in endodermal suberization has been supported by genetic studies. A dramatic decrease in suberin formation is shown in ABA-deficient (*aba2*) and signaling (*abi3*, *abi4*, *abi5*) mutants, as well as when ABA signaling is suppressed in the endodermis [76]. ABA-dependent suberin deposition is mediated by *MYB41-MYB53-MYB92-MYB93*, which are expressed in the endodermis and induced by ABA. A quadruple *myb41 myb53 myb92 myb93* mutant shows a nearly complete lack of endodermal root suberin, which could not be rescued by application of ABA [159], whereas overexpression of some MYB TFs results in ectopic suberin accumulation [156,160] (Figure 2). ABA-induced suberin deposition also requires the auxin-regulated GDSL lipases, some of which have been proposed to be involved in suberin degradation while other in ABA-mediated suberin synthesis [159].

While ABA promotes suberin synthesis, ethylene triggers its degradation, as demonstrated by the enhanced deposition of suberin in the ethylene insensitive mutant, *etr1*, and reduced suberization in the constitutive ethylene signaling mutant, *ctr1* [76]. ABA can partially restore suberization in *ctr1*, suggesting ABA and ethylene signaling may intersect in regulating suberin deposition [76]. A suberin loss-of-function mutant (*suberman/myb39*) and overexpression line show differential expression of ABA and ethylene signalling genes, which further support ABA and ethylene crosstalk in suberin formation in Arabidopsis [161] (Figure 2).

Depending on the availability of nutrients, suberization can be enhanced or decreased, and this process is under hormonal regulation. Under iron (Fe), manganese (Mn) or zinc (Zn) deficiency, ethylene signalling is induced to inhibit endodermal suberization, whereas deficiencies in sulfur (S), potassium (K) or salt stress activate suberin deposition through ABA signalling [76]. Seed yield, rosette size and weight were reduced in the suberin-deficient mutants [76], phenotypes that are also shown by ABA-deficient mutants, suggesting that ABA regulation of plant growth may be in part linked to its ability to regulate suberin deposition, and thus water and nutrient uptake or retention. Interestingly, using ABA deficient (*aba2*) and signaling (*abi4*) mutants, as well as endodermal-specific suppression of ABA signaling, a recent study highlighted the role of the microbiome in modulating suberin deposition, nutrient uptake and plant growth by suppressing ABA responses [162]. Overall, ABA plays direct and indirect roles in the regulation of suberin biosynthesis, accumulation, and deposition in the endodermis in various stressed and non-stressed conditions.

## 4. Conclusions and Future Directions

A considerable amount of research has been conducted to address the role of ABA in regulating stress responses in plants. However, less is known about the role of ABA in modulating plant growth and developmental processes. Here, we reviewed genetic evidence that supports ABA acting as both a positive and negative regulator of growth. Biphasic ABA responses are often observed in which low doses of ABA promote growth, while high doses inhibit it [5,6]. ABA plays roles in regulating central metabolism and chloroplast biogenesis. Crosstalk between ABA and other phytohormones, including auxin, CK, and ethylene is also important for fine-tuning control over growth. The critical function of basal ABA in promoting growth is to negatively regulate ethylene biosynthesis. This ABA-ethylene interaction also plays a major role in regulating local growth responses. In terms of molecular mechanisms, ABA regulates ethylene biosynthesis via multiple different pathways, including transcriptional and post-translational regulations [163]. For example, ABA can inhibit ethylene biosynthesis through ABI4. ABI4 is shown to bind to the promoter of *ACS* genes and repress their transcription [164]. Auxin and CK pathways have critical roles in regulating cell cycle progression of roots and shoots, and are known to interact with ABA signaling components. Overall, phytohormone crosstalk can be positive or negative depending on the tissue and developmental stage being assessed, highlighting a need for specificity and precision in future studies.

ABA also plays key roles in the determination of cell fate and development of tissues that are involved in transpiration and gas exchange, as well as water and nutrient transport, by regulating the biosynthesis of cutin, waxes and suberin. Specifically, genetic evidence supports a negative role for ABA in stomata development, and a positive role in the deposition of shoot epidermal waxes and cutin. ABA also positively regulates suberin deposition in the root endodermis and xylem differentiation in the vasculature of various higher plants. While ABA promotes xylem development non-cell autonomously, it acts in the endodermis to promote suberin deposition in a cell autonomous manner [143,144,152]. ABA works synergistically with auxin to promote xylem differentiation, while acting antagonistically with ethylene in suberin deposition. ABA’s role in modulating plant growth and stress response is directly linked to its function in regulating chloroplast biogenesis, which affects metabolism including processes required for the development of cells and tissues that are pivotal for nutrient uptake and transpiration.

Despite the data available describing ABA’s role in growth and development, there are still outstanding questions to be addressed in the future. Although basal ABA is required for the development of specific tissues, the action of ABA in these cell and tissue types are unknown. Cell- or tissue-type specific transcriptomic studies coupled with ChIP-seq should help elucidate regulatory circuits and identify direct targets of hormone signaling components in different cell and tissue types. This should also aid in understanding mechanisms of cell and non-cell autonomous signaling and is critical to uncover other players as well as evidence of hormone crosstalk. These studies should be accompanied by metabolomic analyses in different tissues and at different stages of development. Most of the studies discussed here were conducted in seedlings, leaves or roots, however there is a little genetic evidence on the role of basal ABA in the development of other organs or at different stages of development. Lastly, to uncover mechanisms of evolution in ABA-mediated control of growth and development, it is critical that we extend our knowledge to other plant species.

## Figures and Tables

**Table 1:** Growth and developmental phenotypes of ABA metabolism and signaling mutants described in this article. Growth-related and development-related phenotypes of mutants are highlighted in green and yellow, respectively.

Species	Mutant	Gene Function	Growth		Development	
			Phenotypes	Ref.	Phenotypes	Ref.
*A. thaliana*	***aba1***	ZEP	• Reduced fresh weight • Stunted root growth • Short hypocotyls after dark germination • Decreased photosystem activity • Increased number of chloroplasts • Enhanced hyponastic response • Disturbed root meristem maintenance	[8,10,59,60,61,62]	• Increased stomatal index or density	[63,64]
*A. thaliana*	***nced3 nced5***	NCED	• Reduced rosette diameter	[65]	• Increased stomatal density • Failed to decrease stomatal density in elevated CO_2_	[66]
*A. thaliana*	***aba2***	xanthin dehydrogenase	• Stunted growth • Glucose insensitivity • Enhanced hyponastic response • Enhanced ethylene evolution rate • Enhanced leaf emergence on plates •Disturbed root meristem maintenance	[59,61,67,68,69,70]	• Increased stomatal index and density • Increased permeability of cuticle and ROS production • Thinner cell walls • Decreased lignin and crystalline cellulose content • Decreased suberin formation • Discontinued metaxylem strands	[71,72,73,74,75,76]
*A. thaliana*	***aba3***	Moco sulfurylase	• Reduced fresh weight • Enhanced hyponastic response • Disturbed root meristem maintenance	[59,61,77]	• Increased stomatal index or density • Failed to decrease stomatal density in elevated CO_2_ • Discontinued metaxylem strands • Increased permeability of cuticle and ROS production	[66,71,73,78]
*A. thaliana*	***aao3***	Abscise aldehyde oxidase	• Stunted growth	[79]		
*A. thaliana*	***pyr1 pyl1 pyl2 pyl4; pyl112458; pyl duodecuple***	PYR/PYL/RCAR	• Reduced plant height • Reduced rosette diameter • Reduced fresh weight • Stunted root growth	[35,37]	• Fail to decrease stomatal density in elevated CO_2_ • Increased cuticle permeability • Fail to induce ectopic protoxylem in presence of ABA	[66,73,74,78]
*A. thaliana*	***hab1 abi1-2 abi2-2; hab1 abi1-2 pp2ca*** **(triple *pp2c*)**	PP2C	• Reduced fresh weight • Reduced leaf surface area • Stunted root growth	[80]		
*A. thaliana*	***snrk2.2/2.3/2.6*** **(*snrk2* triple)**	SnRK2	• Reduced fresh weight • Reduced stem height • Reduced leaf surface area • Stunted root growth • Enhanced leaf emergence on plates	[32,33,67]	• Increased cuticle permeability • Thinner cell walls • Decreased lignin and crystalline cellulose content	[78,81]
*A. thaliana*	***abi1-1***	PP2C	• Enhanced hyponastic response •Disturbed root meristem maintenance	[59,61]	• Increased stomatal index • Increased cuticle permeability • Fail to induce ectopic protoxylem in presence of ABA	[72,73,74,78]
*A. thaliana*	***abi2-1***	PP2C	•Disturbed root meristem maintenance	[61]	• Increased stomatal index	[72]
*A. thaliana*	***abi3***	ABI3	• Enhanced hyponastic response •Disturbed root meristem maintenance	[59,61]	• Decreased suberin formation	[76]
*A. thaliana*	***abi4***	ABI4	• Glucose insensitivity • Enhanced lateral root formation and growth	[70,82]	• Decreased suberin formation	[76]
*A. thaliana*	***abi5***	ABI5	•Disturbed root meristem maintenance	[61]	• Decreased suberin formation	[76]
*A. thaliana*	***cyp707a1 cyp707a3***	CYP707A			• Decreased stomatal index and density	[72]
*A. thaliana*	***atbg1 atbg2***	AtBG			• Increased stomatal density	[83]
*S. lycopersicum*	***hp3***	ZEP	• Increased number of chloroplasts	[10]		
*S. lycopersicum*	***notabilis***	NCED	• Reduced leaf surface area • Reduced dry leaf weight • Short hypocotyls after dark germination	[84,85,86,87,88]	• Increased stomatal index and density • Altered composition of cutin and cutilcilar waxes • Decreased level of cutin	[89,90,91]
*S. lycopersicum*	***flacca***	Moco sulfurylase	• Reduced leaf surface area • Reduced dry leaf weight • Reduced fruit size • Reduced assimilation rate • Increased number of chloroplasts • Enhanced ethylene evolution rate	[10,84,85,86,88,92,93]	• Increased stomatal index and density • Altered composition of cutin and cutilcilar waxes • Decreased level of cutin	[89,90,91]
*S. lycopersicum*	***sitiens***	abscisic aldehyde oxidase	• Stunted growth • Stunted hypocotyls after dark germination • Enhanced lateral root formation • Enhanced assimilation rate • Increased number of chloroplasts	[10,84,87,88,92,93,94,95]	• Inceased stomatal index and density • Altered composition of cutin and cutilcilar waxes • Decreased level of cutin	[88,96,97]
*P. sativum*	***wilty***	xanthin dehydrogenase	• Enhanced lateral root formation	[94]		
*Z. mays*	***vp5***	PDS	• Stunted root growth • Enhanced ethylene evolution rate	[98]		
*O. sativa*	***oszep***	ZEP	• Enhanced shoot growth after submergence	[99]		
*O. sativa*	***osaba2***	xanthin dehydrogenase	• Enhanced stem height • Overaccumulation of ROS	[13]		
*O. sativa*	***ospyl1/4/6***	PYR/PYL/RCAR	• Enhanced panicle length • Enhanced fresh weight	[36]		
*H. vulgare*	***Az34***	Moco biosynthesis	• Decreased net assimilation rates	[22,93]		

## Data Availability

Not applicable.
